# Combinatorial Treatment of Birinapant and Zosuquidar Enhances Effective Control of HBV Replication In Vivo

**DOI:** 10.3390/v12080901

**Published:** 2020-08-17

**Authors:** Emma Morrish, Liana Mackiewicz, Natasha Silke, Marc Pellegrini, John Silke, Gabriela Brumatti, Gregor Ebert

**Affiliations:** 1 The Walter and Eliza Hall Institute of Medical Research, Melbourne, VIC 3052, Australia; morrish.e@wehi.edu.au (E.M.); mackiewicz@wehi.edu.au (L.M.); nsilke@wehi.edu.au (N.S.); pellegrini@wehi.edu.au (M.P.); silke@wehi.edu.au (J.S.); brumatti@wehi.edu.au (G.B.); 2 Department of Medical Biology, University of Melbourne, Melbourne, VIC 3010, Australia

**Keywords:** HBV, inhibitor of apoptosis proteins, IAP, IAP antagonist, Smac-mimetics, TNF, cell death, MDR1 inhibitor, combination therapy

## Abstract

Chronic hepatitis B virus (HBV) infection remains a global health threat and affects hundreds of millions worldwide. Small molecule compounds that mimic natural antagonists of inhibitor of apoptosis (IAP) proteins, known as Smac-mimetics (second mitochondria-derived activator of caspases-mimetics), can promote the death of HBV-replicating liver cells and promote clearance of infection in preclinical models of HBV infection. The Smac-mimetic birinapant is a substrate of the multidrug resistance protein 1 (MDR1) efflux pump, and therefore inhibitors of MDR1 increase intracellular concentration of birinapant in MDR1 expressing cells. Liver cells are known to express MDR1 and other drug pump proteins. In this study, we investigated whether combining the clinical drugs, birinapant and the MDR1 inhibitor zosuquidar, increases the efficacy of birinapant in killing HBV expressing liver cells. We showed that this combination treatment is well tolerated and, compared to birinapant single agent, was more efficient at inducing death of HBV-positive liver cells and improving HBV-DNA and HBV surface antigen (HBsAg) control kinetics in an immunocompetent mouse model of HBV infection. Thus, this study identifies a novel and safe combinatorial treatment strategy to potentiate substantial reduction of HBV replication using an IAP antagonist.

## 1. Introduction

Many pathogens, including hepatitis B virus (HBV), effectively manipulate tumor necrosis factor (TNF) receptor pathways and control molecular processes within host cells to evade antiviral immune responses and promote their propagation [1]. We have shown that HBV-expressing hepatocytes display high sensitivity to TNF signaling, with increased expression of both TNF and TNF receptor 1 (TNFR1), warranting the investigation of how the TNFR1 pathway can be therapeutically leveraged for the elimination of HBV infected cells [2].

Cellular inhibitor of apoptosis (cIAP) proteins are major regulators of the extrinsic TNFR cell death pathway, orchestrating cell survival and cell death [3,4]. Natural IAP antagonists, such as the second mitochondria-derived activator of caspases (Smac/DIABLO), can sensitize cancers to cell death. This has led to the pharmaceutical development of small molecule peptide-mimetic IAP antagonists (Smac-mimetics) [5]. The Smac-mimetic birinapant has predominantly been investigated for the treatment of cancer [6,7,8,9]. However, the observation that cIAPs limit TNF-mediated apoptosis and that Smac-mimetics sensitize cells to TNF induced cell death [10,11,12] led to the idea that these drugs might also be used for therapeutic applications in infectious diseases. Supporting this approach, studies by our group have shown that genetic loss of cIAPs, or their degradation through birinapant treatment, promoted death of HBV infected hepatocytes. Furthermore, birinapant therapy enhanced HBV-DNA serum elimination in an immunocompetent mouse model of HBV infection [2,13]. These findings were not limited to birinapant, as the Smac-mimetic APG-1387 (Ascentage Pharma Group) was also able to kill HBV infected liver cells [14]. Both studies prompted the initiation of clinical trials with birinapant (NCT02288208) and APG-1387 (NCT03585322) in chronic HBV infected patients. Importantly, we applied an identical strategy using Smac-mimetics to eliminate liver stage malaria parasite infection, demonstrating their therapeutic potential in other infectious diseases [15]. Despite the initial promising results for the use of Smac-mimetics for infectious disease therapy, adverse effects have been observed when these drugs are used at higher concentrations. Thus, low dose Smac-mimetic therapeutic strategies, that can specifically kill HBV-replicating cells sparing healthy organs, are of interest.

We have recently identified birinapant as a substrate of the multidrug resistance protein 1 (MDR1) efflux pump [16]. MDR1 (also known as P-gp or ABCB1) is a member of the ATP-binding cassette (ABC) transporter family and these transporters actively pump out toxic substrates from within the cell into the extracellular space, protecting cells from possible toxicity [17]. Co-treatment of birinapant with the clinical third generation specific MDR1 inhibitor zosuquidar (LY335979) [18] potentiated the efficacy of birinapant killing in malignant disease [16]. MDR1 is widely expressed in the liver including in hepatocytes [19], suggesting that co-treatment with MDR1 inhibitor zosuquidar may enhance the therapeutic anti-HBV activity of birinapant.

## 2. Materials and Methods

### 2.1. Reagents

Birinapant was purchased from SelleckChem (Houston, TX, USA) and zosuquidar trihydrochloride from MedChemExpress (Monmouth Junction, NJ, USA). Recombinant human TNF was produced in-house at the Walter and Eliza Hall Institute of Medical Research (WEHI).

### 2.2. Tissue Culture and Viability Assays

Human HepG2 cells were cultured in Roswell Park Memorial Institute (RPMI) medium from Gibco (Waltham, MA, USA), supplemented with 10% fetal calf serum (FCS), at 37 °C in a 10% CO_2_ humidified atmosphere. Each IncuCyte viability assay was performed in triplicate by seeding 0.25 × 105 HepG2 cells per well in a CellCarrier-96 plate (PerkinElmer, Waltham, MA, USA). Cells were cultured with propidium iodide (PI) 3 h prior to treatment. Using IncuCyte S3 2018A software (Essen BioScience, Ann Arbor, MI, USA), two images per well were taken and the number of PI positive cells calculated. Time course assays for Western blotting were performed by seeding 5 × 105 HepG2 cells per well in a six-well plate. Cells were pre-treated with zosuquidar for 20 min prior to addition of TNF and birinapant. The chemotherapy drug cisplatin was used as a positive control to induce cell death as previously reported [20]. At the indicated time points, cells were harvested, pelleted and frozen at −20 °C overnight.

### 2.3. In Vivo Model Studies of Hepatitis B Virus (HBV)

The WEHI Animal Ethics Committee approved all in vivo experiments (Project No. 2017.016). Murine models of HBV transduction of infection, analysis of alanine aminotransferase (ALT) and serial serum HBV-DNA analysis were carried out as previously described [2,13]. HBV infection was induced in immunocompetent male C57BL/6 mice through hydrodynamic injection (HDI) of an HBV encoding plasmid via the tail vein as previously reported [2,21,22]. Zosuquidar was prepared in 2% D-mannitol, 0.015% glycine in sterile H_2_0 and diluted in saline solution, phosphate-buffered saline (PBS). Five days following induction of infection, mice were pre-treated by intraperitoneal (IP) injection with zosuquidar (5 or 25 mg/kg) for three days. Five hours following the last dose of zosuquidar, birinapant (3 mg/kg) was delivered by IP injection. For ALT analysis, animals were culled 16 h later. The same experimental plan was performed with uninfected animals. Serum from cardiac bleeds was isolated and analyzed for ALT using a Cobas e411 Analyzer (Roche, Basel, Switzerland) according to the manufacturer’s instructions. 

For serial serum HBV-DNA analysis, mice were IP injected with zosuquidar three times a week (5 or 25 mg/kg) and birinapant once a week (10 mg/kg) for three weeks. Mice were submandibular bled once a week and serum was analyzed for HBV-DNA and HBV surface antigen (HBsAg) content. Briefly, the High Pure Viral Nucleic Acid Kit (Roche) or the Invisorb Virus DNA HTS 96 Kit (Stratec, Birkenfeld, Germany) was used to extract viral DNA from prepared serum and real-time PCR was used to quantify DNA load. The DNA Amplification SYBR Green Kit (Roche) on a LightCycler 480 II Machine (Roche) with Absolute Quantification Software (Roche) was used to further quantify DNA load. Serial dilutions of pAAV-HBV1.2 were used as the standard. Primers were HBV1745fw (GTTGCCCGTTTGTCCTCTAATTC) and HBV1844rev (TGAGGGAAACATAGAGTTGCCTTGA). The limit of detection of serum HBV-DNA was 500 copies/mL.

Quantification of serum HBsAg was performed by isolating and diluting serum 1:200 in PBS and analyzed using a Cobas e411 Analyzer (Roche) according to the manufacturer’s instructions. Detection threshold cut off was 2 IU/mL. 

### 2.4. Preparation of Mouse Livers for Western Blotting

Immediately following death, sample tissue from mouse livers was harvested and snap frozen using liquid nitrogen and stored at −20 °C. Samples were lysed in Cell Lysis Buffer (20 nM Tris HCL pH 7.5, 135 mM NaCl, 1.5 mM Mg_2_Cl, 1 mM EGTA (ethylene glycol-bis(2-aminoethylether)-*N,N,N′,N′*-tetraacetic acid), 1% Triton X-100, 10% Glycerol, 1× final concentration of Protease Cocktail (Roche) and 1× final concentration of PhosSTOP (Roche). Samples were spun at 15,000× *g* for 5 min at 4 °C and the concentration of protein in soluble supernatants was determined by bicinchoninic acid (BCA) assay (Thermo Fisher, Waltham, MA, USA) according to manufacturer’s instructions.

### 2.5. Western Blot Protein Analysis

HepG2 cell pellets or mouse liver samples were prepared in 1× SDS (Sodium dodecyl sulfate) buffer (50 mM tris-HCl (pH 6.8), 2% SDS, 10% glycerol, and 2.5% b-mercaptoethanol) and boiled for 7 min at 100 °C. Samples were loaded onto a 10–12% SDS-polyacrylamide gel and transferred to a nitrocellulose membrane. Membranes were blocked for 1 h at room temperature in 5% (*w/v*) non-fat milk in Tris-buffered saline Tween-20 buffer (TBS-T) (0.1% Tween, 150 mM NaCl, 10 nM Tris-HCl, pH 7.5). Membranes were rinsed in TBS-T prior to incubation with primary antibodies; anti-MDR1 monoclonal antibody (mAb) (Abcam, Cambridge, United Kingdom, clone EPR1036457, Cat#ab170904, Lot#GR21757634), anti-cIAP1 mAb (made at WEHI, clone 1E1-1-12), anti-cIAP2 mAb (made at WEHI, clone 15C8-12), cleaved caspase (Cl Casp) 8 mAb (Cell Signaling Technology, Danvers, MA, USA, clone Asp387-D5B2, Cat#8592, Lot#04/2019), Cl Casp3 mAb (Cell Signaling Technology, Danvers, MA, USA, clone D175, Cat#9661L, Lot#06/2018) and anti-Actin mAb (Sigma, St. Louis, MO, USA, clone AC-15 Ref#A-1978) for 24−72 h at 4 °C. Immunoblots were probed with the specific secondary (anti-rabbit IgG, anti-mouse IgG or anti-rat IgG) antibody conjugated to horseradish peroxidase (Southern Biotech, Birmingham, AL, USA). Bound antibodies were visualized by Chemidoc Touch Imaging System (Bio-Rad, Hercules, CA, USA) using enzyme-linked chemiluminescence (ECL) Immobilion Western (Millipore, Burlington, MA, USA).

### 2.6. HBcAg Immunohistochemistry

Mouse livers were collected 16 h post treatment, perfused ex vivo with PBS, submerged in 10% buffered formalin and embedded in paraffin. Four–six mM tissue sections were prepared and stained with anti-HBcAg antibody (mouse anti HBcAg; 1:500; Abcam ab8637). Slides were scanned using a Panoramic SCAN II scanner (3D Histech, Budapest, Hungary). Images were analyzed in CaseViewer 2.8 (3D Histech).

### 2.7. Statistical Analysis

Statistics were calculated using Prism 7/8 (GraphPad) software and are described in detail in the Figure legends. *p* * < 0.05, *p* ** < 0.01.

## 3. Results

### 3.1. MDR1 Inhibition Enhances Birinapant-Mediated Killing of HepG2 Cells

**Figure 1:**
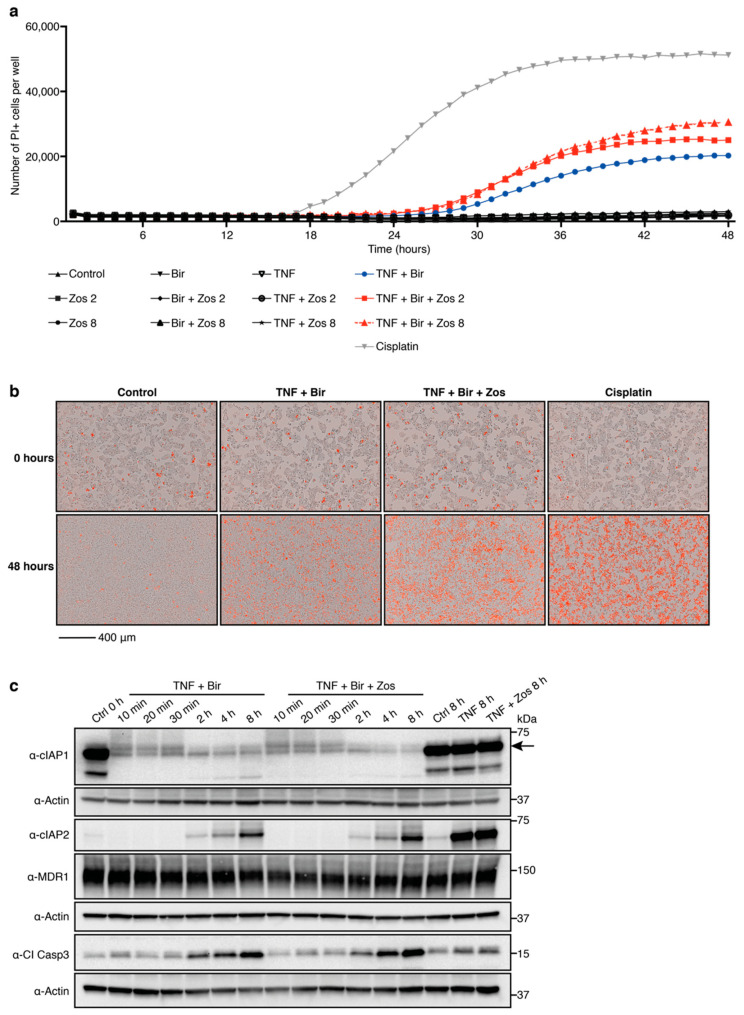
Multidrug resistance protein 1 (MDR1) inhibition enhances birinapant-mediated killing of HepG2 cells. (**a**,**b**) HepG2 cells were cultured with propidium iodide (PI) for 3 h prior to the addition of birinapant (bir) 10 μM ± zosuquidar (zos) 2 or 8 μM ± tumor necrosis factor (TNF) 200 ng/mL; treatment with cisplatin 80 μM was used as a positive control. Analysis of cell death kinetics were performed on an Essen IncuCyte S3. (**a**) Number of PI positive cells per well over 48 h. Plotted is the mean of 3 biological repeats and is representative of 3 independent experiments. (**b**) Visual images of HepG2 cells at 0 and 48 h. One representative experiment of 3 independent experiments is shown, with 3 biological repeats per condition. Red cells are PI positive. Scale bar, 400 μm. (**c**) HepG2 cells were treated with birinapant 10 μM ± zosuquidar 2 μM ± TNF 200 ng/mL for the indicated times. Whole cell lysates were probed with the indicated antibodies. Actin was used as a loading control. Representative of 3 independent experiments. Cl, cleaved; Casp, caspase.

We have shown that the combination treatment of birinapant with zosuquidar potentiates Smac-mimetic-mediated killing of hematopoietic malignancies [16]. Hepatocytes have also been reported to express MDR1 [19], therefore, we investigated whether the MDR1 inhibitor zosuquidar could synergize with birinapant to kill the human liver cancer cell line HepG2. Birinapant did not induce HepG2 cell death after 48 h of treatment either as a single agent or in combination with zosuquidar (bir + zos). Cisplatin treatment, however, killed HepG2 cells as previously described [20] (Figure 1a). Because the killing efficacy of Smac-mimetics is dependent on a cell’s autocrine TNF/TNFR1 signaling, which is limited in HepG2 cells [23], we hypothesized that addition of exogenous TNF would increase birinapant-mediated cell death in HepG2 cells. As expected, addition of TNF (TNF + bir) sensitized HepG2 cells to birinapant treatment, and this was further enhanced with the addition of zosuquidar (TNF + bir + zos) (Figure 1a,b). Analysis of HepG2 cells treated with TNF + bir or the combination of TNF + bir + zos, showed that, at the concentrations and time points tested, there was comparable degradation of cIAP1, cIAP2 and activation of Cl Casp3 in both treatment groups (Figure 1c). Together these data indicate the potential for zosuquidar adjuvant therapy to enhance birinapant-mediated apoptosis in cells of liver origin.

### 3.2. Combination Treatment with Birinapant and Zosuquidar Is Safe and Increases Death of Hepatocytes in the Liver of HBV-Replicating Mice

**Figure 2:**
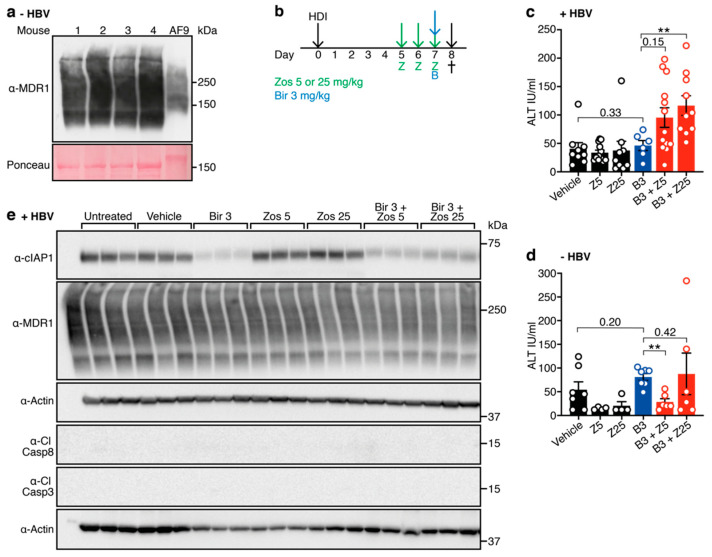
Birinapant in combination with zosuquidar enhances death of hepatitis B virus (HBV)-replicating hepatocytes in C57BL/6 mice. (**a**) MDR1 is expressed in the liver of C57BL/6 mice. AF9—murine MLL-AF9 leukemic sample as a positive control. Ponceau was used as a loading control. (**b**) C57BL/6 mice were hydrodynamically injected (HDI) with HBV encoding plasmid (day 0) and preloaded with zosuquidar (zos) 5 mg/kg or 25 mg/kg for three days (day 5, 6 and 7) prior to ± birinapant (bir) 3 mg/kg treatment (day 7). Mice were culled (†) 16 h following birinapant treatment (day 8) and cardiac blood was analyzed for alanine aminotransferase (ALT) levels. ALT levels in (**c**) HBV replicating (*n* = 6−13, 3 independent experiments) and (**d**) naïve mice (*n* = 4−7, 2 independent experiments) treated with zosuquidar (Z) 25 mg/kg ± birinapant (B) 3 mg/kg. (**e**) Western blot analysis of total liver cell lysates from mice that replicate HBV (day 8) were probed with the indicated antibodies. Actin was used as a loading control. Cl, cleaved; Casp, caspase. Graphs show mean ± standard error of the mean (SEM); ** *p* < 0.01 (nonparametric Mann-Whitney test).

MDR1 is expressed in the livers of C57BL/6 mice [19] (Figure 2a) and we have previously shown that a 30 mg/kg dose of birinapant can synergize with endogenously expressed TNF to kill HBV-expressing hepatocytes in an immunocompetent mouse model of HBV replication [13]. This suggests that the combination of birinapant and zosuquidar may be effective for the treatment of HBV infection, allowing lower doses of birinapant to be used for the same antiviral effect. To test this, we treated animals with birinapant (10 mg/kg), either alone or in combination with different doses of zosuquidar (5 mg/kg or 25 mg/kg) (Figure 2b). A sensitive measurement of hepatocyte death is ALT level. We have shown that high ALT-levels correlate with Smac-mimetic mediated induction of hepatocyte death in a dose dependent fashion [13,15]. The combination of birinapant + zosuquidar treatment was well tolerated in both HBV-replicating and healthy uninfected animals, with no signs of distress or discomfort observed. Interestingly, HBV-replicating animals showed unchanged ALT levels upon birinapant single-agent treatment, but ALT levels were increased in a dose dependent fashion by the addition of zosuquidar (Figure 2c). Most importantly, no increase in ALT levels was observed in uninfected healthy animals treated with the same combination treatment (Figure 2d). Despite obvious loss of cIAP1, we did not detect cleaved caspase 8 or 3 in any of the samples tested (Figure 2e). Together these results indicate that drug combination-mediated killing is specific towards HBV-replicating hepatocytes, resulting in a two-fold increase of ALT levels.

### 3.3. Birinapant and Zosuquidar Combination Therapy Specifically Kills HBV Expresisng Cells in the Liver of Mice

**Figure 3:**
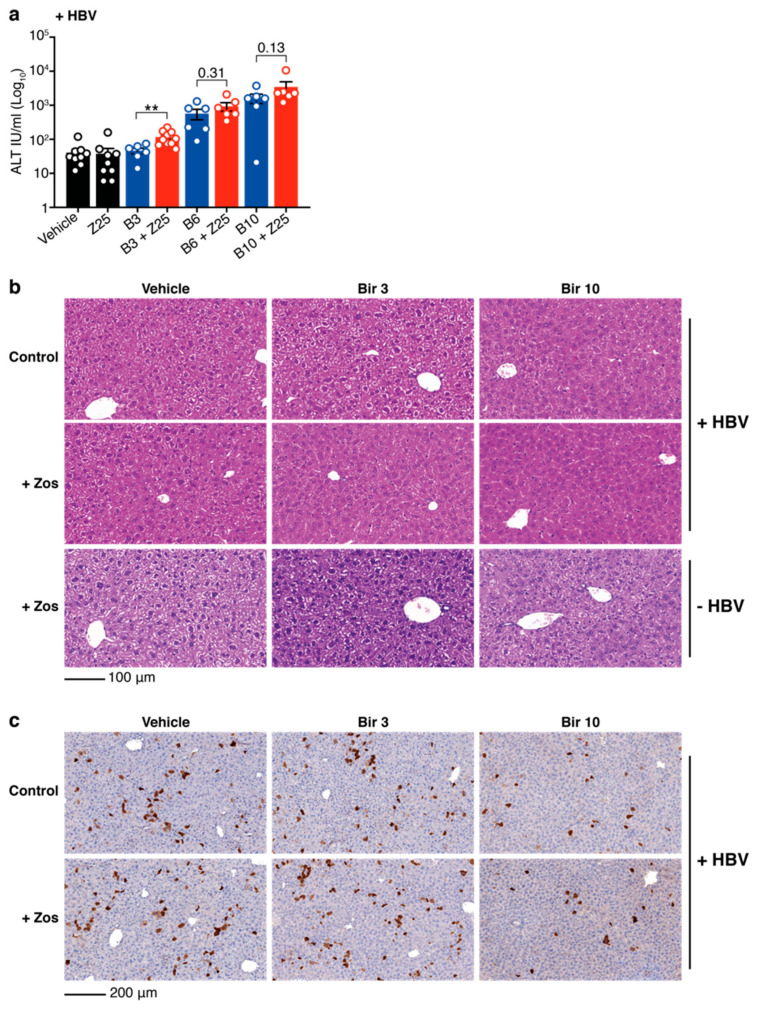
Birinapant plus zosuquidar combination therapy specifically reduces HBV positive cells in the liver of HBV-replicating C57BL/6 mice. (**a**) ALT levels in the serum of HBV-replicating mice treated as indicated at day 8 (see timeline Figure 2b) with zosuquidar 25 mg/kg ± birinapant 3, 6 or 10 mg/kg. Birinapant (B3) and birinapant + zosuquidar (B3 + Z25) data is reiterated from Figure 2c. (**b**) Hematoxylin and eosin (H&E) staining of livers from HBV-replicating and naïve mice at day 8 and treated as indicated. Scale bar, 100 μm. (**c**) HBV core antigen (HBcAg) staining of livers from HBV-replicating mice at day 8 and treated as indicated. Scale bar, 200 μm. Images are representative of *n* = 4−8 mice per group. Graph shows mean ± SEM; ** *p* < 0.01 (nonparametric Mann-Whitney test).

Although a significant increase in ALT is observed, the levels are limited. One possibility to explain the low levels of ALT and the absence of activated caspases is that, at the drug doses tested, the combination treatment induces limited killing of HBV positive hepatocytes. To test whether increased concentrations of birinapant might target more HBV-replicating cells and induce higher levels of ALT, HBV-expressing mice were treated with 6 mg/kg and 10 mg/kg of birinapant in the presence or absence of 25 mg/kg of zosuquidar. ALT levels were tested 16 h after birinapant treatment in the serum of mice. Increasing concentrations of birinapant alone enhanced the release of ALT from the liver and addition of zosuquidar incrementally increased these levels, albeit only to significant values at the lowest concentration of birinapant (Figure 3a). Hematoxylin and eosin (H&E) staining of livers confirmed our previous findings [15] that birinapant up to 10 mg/kg alone and in combination with zosuquidar 25 mg/kg causes no off-target toxicity or killing of healthy liver cells in HBV-replicating and naïve animals (Figure 3b). To determine the proportion of HBV-replicating liver cells, and the capacity of the combination treatment to specifically kill these cells, livers from animals at day 8 (16 h after birinapant treatment) were analyzed for hepatitis B virus core protein (HBcAg) expression by immunohistochemistry. We found that zosuquidar 25 mg/kg in combination with a single dose of birinapant at higher concentrations (10 mg/kg) can more effectively kill core protein positive HBV-replicating liver cells compared to birinapant 3 mg/kg treatments (Figure 3c). Overall, these data suggest that MDR1 inhibition might be used to enhance the antiviral efficacy of birinapant to specifically target and kill HBV-replicating hepatocytes without increasing toxicity.

### 3.4. The MDR1 Inhibitor Zosuquidar Enhances the Efficacy of Birinapant to Diminish HBV Replication In Vivo

**Figure 4:**
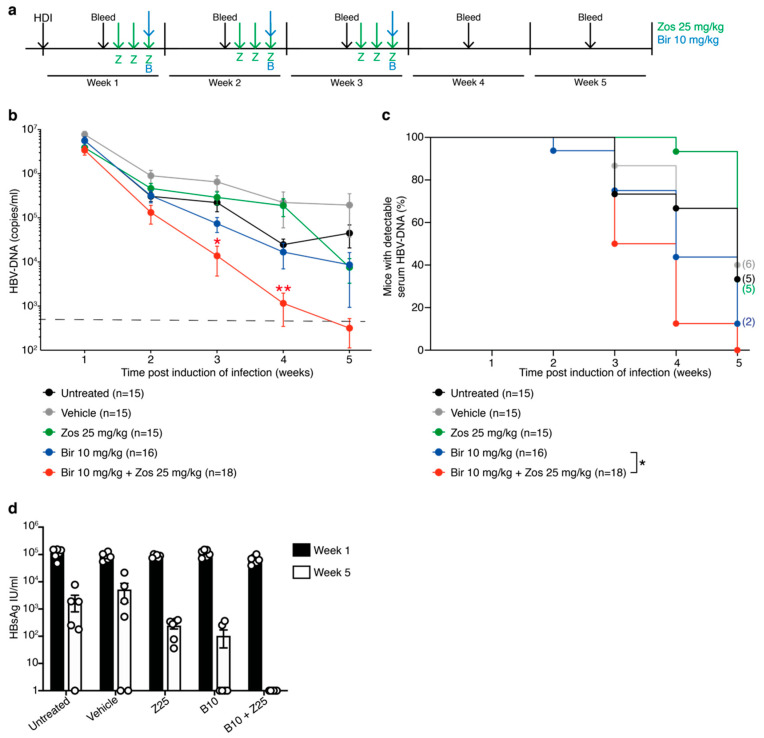
Zosuquidar enhances the efficacy of birinapant to eliminate HBV replication in C57BL/6 mice. (**a**) Long-term analysis treatment scheme of HBV-replicating C57BL/6 mice by HDI and preloaded with 25 mg/kg of zosuquidar (green arrows) for three days prior to ± 10 mg/kg of birinapant (blue arrows). Treatment strategy was repeated for three weeks. Animals were bled (black arrows) weekly prior to treatment and serum HBV-DNA levels analyzed. (**b**) Animals were treated as described in Figure 4a and serum HBV-DNA levels analyzed by qRT-PCR (*n* = 15−19); pooled data of two independent experiments, dashed line indicates assay detection limit of 500 HBV-DNA copies/mL (**c**) Proportion of mice with detectable serum HBV-DNA (*n* = 15−19, pooled data of two independent experiments). Number of mice with detectable HBV-DNA at week 5 is indicated in brackets on the graph; *p* values were calculated using a Log-rank (Mantel-Cox) test comparing birinapant and birinapant + zosuquidar. (**d**) HBV surface antigen (HBsAg) levels in serum of mice at weeks 1 and 5 post induction of infection. Graphs show mean ± SEM; * *p* < 0.05; ** *p* < 0.01 (Figure 4b, nonparametric Mann-Whitney test; Figure 4c, log-rank Mantel–Cox test).

Given the encouraging results in the short-term assays with the combination of birinapant and zosuquidar, we investigated whether these findings could be translated to the more clinically relevant clearance of HBV-DNA in our murine HBV model. We have previously demonstrated that weekly doses of birinapant at 30 mg/kg rapidly induces control of HBV replication in mice, whereas doses of 10 mg/kg birinapant in comparison revealed delayed efficacy [13]. However, based on the increased cell death in animals treated with 10 mg/kg birinapant in combination with zosuquidar (Figure 3), C57BL/6 mice were treated with 25 mg/kg of zosuquidar for three days followed by 10 mg/kg birinapant after induction of infection by HDI. This treatment regimen was used for three weeks and HBV-DNA serum levels determined weekly (Figure 4a). Analysis of HBV-DNA levels revealed no difference in reduction rates between untreated, vehicle and zosuquidar single agent treated animals (Figure 4b,c). However, combined treatment of birinapant and zosuquidar significantly reduced the time taken for animals to diminish HBV virus compared to 10 mg/kg of birinapant single-agent treatment (Figure 4b,c). These results were accompanied by a greater reduction in HBsAg, in the serum of mice treated with combination therapy in comparison to birinapant or zosuquidar single drug treatment (Figure 4d). These data therefore support the idea that an MDR1 inhibitor can safely increase the efficacy of birinapant to mediate effective abolishment of HBV replication in preclinical model of HBV infection.

## 4. Discussion

HBV infection remains a global health threat, with around 45% of the world’s population living in areas of high prevalence and an estimated 257 million people suffering from chronic disease [24]. In primary self-limiting HBV infection, effective immune responses clear the virus; however, persistent chronic HBV infection with continuous virus production leads to liver inflammation, increasing the risk of liver cirrhosis and liver cancer development [25]. Current standard of care antiviral therapies comprising of HBV polymerase inhibitors, such as entecavir and tenofovir, efficiently control viral replication but cannot eliminate the virus and have to be taken lifelong. Therefore, novel strategies that overcome chronic HBV infection and eliminate the virus are urgently required.

Our prior work demonstrating that birinapant efficiently promotes the clearance of HBV in vivo [2,13] led to clinical trials of birinapant for the treatment of chronic HBV patients (NCT02288208). To be approved for the treatment of non-life-threatening conditions, drugs must, understandably, show no toxicity. Unfortunately, the mild and reversible toxicities associated with birinapant halted clinical trials into using birinapant to eradicate HBV. This drove us to test whether the anti-HBV activity of birinapant could be safely potentiated. Our recent finding that birinapant is a substrate of MDR1, and that MDR1 inhibitors can safely enhance birinapant therapy in malignant disease [16], prompted us to determine whether the third generation specific MDR1 inhibitor zosuquidar could be used to safely increase the efficacy of birinapant treatment of HBV infection.

MDR1 has been reported to be expressed in hepatocytes [19] and we confirmed that inhibition of MDR1 can enhance birinapant-mediated killing of HepG2 liver cells in the presence of TNF in vitro. After we verified hepatocellular MDR1 expression in C57BL/6 mice, we investigated the ability of birinapant in combination with zosuquidar to induce apoptosis of HBV positive cells in an in vivo model of HBV infection. We have previously shown that this TNF mediated process relies on functional immune signaling networks [2,13] and therefore established HBV replication in immunocompetent C57BL/6 mice by HDI [2,21,22]. The model facilitates full HBV replication in the liver of animals transiently for weeks and months and, although formation of episomal cccDNA seems to be lacking or minimal, sub-genomic fractions of the viral genome integrated into the host genome of transduced hepatocytes could contribute to viral protein expression. However, released viral particles do not re-infect hepatocytes due to the lack of the essential HBV surface receptor [26].

Using this model, we measured acute HBV liver cell damage by assessing ALT levels in the serum of mice following treatment. We have previously shown that ALT levels in HBV infected animals correlate well with therapeutic effect [13,15]. Importantly, the combination treatment did not induce an increase in ALT levels in uninfected healthy animals and we did not observe any obvious liver specific off-target toxicity or liver cell damage in naïve and HBV-replicating animals, supporting the positive safety profile of this treatment. Non-specific and indiscriminate induction of apoptosis would lead to apparent hepatic destruction and logarithmically higher ALT levels, as we have shown previously by inducing non-specific, TNF mediated liver apoptosis using galactosamine (GalN) and lipopolysaccharide (LPS), causing systemic hepatitis [15,27].

Interestingly, although addition of zosuquidar increased birinapant mediated liver cell death and ALT levels in HBV-replicating animals in a dose dependent fashion, we did not observe increased degradation of birinapant’s target proteins, cIAP1 and cIAP2. Due to the inherent limitations of measuring sensitive changes in protein expression in whole organ lysates, in combination with the limited number of hepatocytes replicating HBV in the liver of animals, further studies are required for a better understanding of the molecular mechanism induced by the combination treatment in HBV infection models.

Lastly, we investigated the therapeutic potential of the combinatorial treatment using birinapant and zosuquidar to impact on HBV-replication in mice. Importantly, our results show significantly enhanced control kinetics of HBV-DNA and HBsAg in the serum of mice treated with the combination therapy, compared to single-agent treatments. However, a more detailed analysis of potential adverse effects in other animal organs treated with 30 mg/kg of birinapant [2,13] versus the combination of lower concentrations of birinapant and zosuquidar would be necessary to advance these findings into clinical trials.

Smac-mimetic mediated killing of HBV infected hepatocytes eliminates the HBV genome, regardless of its nature, although the approach is limited by the ability to induce exclusively apoptosis of active HBV replicating hepatocytes, but not replicative inactive liver cells harboring, for example, currently silenced cccDNA. The ability of birinapant to preferentially kill HBV-replicating cells while leaving normal cells intact remains to be fully elucidated but could also reflect their function in regulating the response to infection. In insect cells, IAPs are so critical for cell survival that baculoviruses express viral versions to keep infected cells alive [28]. In mammals, IAPs play many roles but the fact that Smac-mimetics sensitize mammalian cells to TNF induced death clearly suggests that their identification as inhibitor of apoptosis proteins reflects a deeper truth, particularly in an infectious or inflammatory environment. This is emphasized by the fact that healthy MDR1-expressing cells undergo birinapant-mediated degradation of cIAPs, yet these cells are far less susceptible to the cytotoxic effects of birinapant. A possible explanation for the different responses of healthy and HBV infected cells might be that the latter have abnormal expression of TNF pathway related signaling molecules including IAPs, which leads to an increased dependency on the NF-κB and/or the TNFR1 signaling pathways. Hence, Smac-mimetic mediated preferential killing and specificity towards actively HBV expressing hepatocytes is likely to be driven by their sensitivity due to production of TNF and upregulation of TNFR1 expression [2]. Given that normal, uninfected liver cells are not dying in response to cIAP inhibition, these changes must be overrepresented in HBV infected cells with actively replicating virus. Consistent with this idea, our previous studies in mouse models of HBV highlighted the importance of TNF and TNFR1 expression for the birinapant-mediated clearance of virus from infected liver cells [13]. Importantly, the mechanism of TNF and caspase mediated induction of apoptosis of target cells remains identical in the combinatorial approach of birinapant and MDR1 inhibitor [16].

Although we have demonstrated that birinapant induced death of HBV-replicating hepatocytes [13], it is possible that changes in the liver environment may also contribute to the increased sensitivity and killing of infected cells. Furthermore, targeting host cellular IAPs with Smac-mimetics to eliminate HBV infection limits the possibility of development of treatment resistant HBV mutants, supporting the notion of these host-targeted therapies as highly attractive therapeutic approaches to eliminate chronic HBV infection.

Altogether, our data suggest that combination of birinapant plus the MDR1 inhibitor zosuquidar is a safe and targeted therapy to kill HBV-replicating hepatocytes. Moreover, the observation that zosuquidar can safely potentiate the anti-viral effect of birinapant in an in vivo HBV model opens a way to reduce the dose limiting toxicities previously reported in Smac-mimetic clinical trials for HBV infection. This is an important finding and has the potential to be transferable across a variety of applications, including the usage of birinapant to eliminate liver stage specific infections, such as malaria, and oncogenic diseases.
